# Late resistance to imatinib therapy in a metastatic gastrointestinal stromal tumour is associated with a second KIT mutation

**DOI:** 10.1038/sj.bjc.6601819

**Published:** 2004-04-20

**Authors:** T Wakai, T Kanda, S Hirota, A Ohashi, Y Shirai, K Hatakeyama

**Affiliations:** 1 Division of Digestive and General Surgery, Niigata University Graduate School of Medical and Dental Sciences, 1-757 Asahimachi-dori, Niigata City, 951-8510 Japan; 2 Department of Pathology, Osaka University Medical School, Suita, Osaka, Japan

**Keywords:** c-*kit* gene, gastrointestinal stromal tumours, imatinib, molecular target therapy, secondary resistance

## Abstract

Currently, there are no data on the secondary resistance of gastrointestinal stromal tumours to imatinib. Here, we report a case of metastatic gastrointestinal stromal tumour that relapsed during imatinib therapy. Mutation analysis showed that the imatinib-resistant liver tumour contained two c-*kit* mutations.

Gastrointestinal stromal tumours (GISTs) are the most common mesenchymal tumours of the human gastrointestinal tract. The GISTs are often characterized by expression levels of KIT, a tyrosine kinase, and many tumours contain mutations of the KIT-encoding gene, c-*kit* (Hirota *et al*, 1998). Imatinib is a selective tyrosine kinase inhibitor for BCR-ABL, platelet-derived growth factor receptors, and KIT, and has a dramatic antitumour effect on metastatic GISTs (Demetri *et al*, 2002). Heinrich *et al* (2003) recently found a strong association between clinical response to imatinib and tumour genotype, which has clarified our understanding of the molecular mechanisms underpinning primary resistance to imatinib in GISTs. Resistance to imatinib treatment for GISTs can also occur after the initial clinical response. However, the mechanism for this secondary resistance to imatinib in GISTs remains unknown.

Here, we report a case of resistance to imatinib in a patient with metastatic GIST of gastric origin. The tumour relapsed during imatinib treatment after showing a very good clinical response. We also present a c-*kit* mutation analysis of the primary and metastatic tumours of the patient.

## CASE REPORT

**Figure 1:**
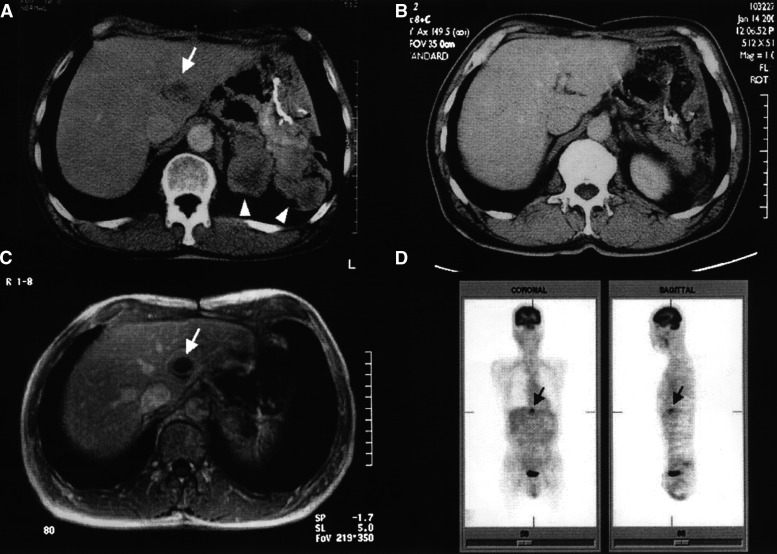
Response to imatinib treatment. Computed tomographic examination before imatinib treatment: a solitary liver metastasis (arrow) and multiple peritoneal metastases (arrowheads) (**A**), CT scan after 6 months of imatinib treatment showing a partial response (**B**), gadolinium-enhanced T1-weighted MRI after 9 months of imatinib treatment showing disease progression only in the liver metastatic deposit (arrow) (**C**), PET scan after 10 months of imatinib treatment. The increased uptake of the tracer [^18^F]FDG was seen only in the liver metastatic deposit (arrow) (**D**).

A 64-year-old Japanese man underwent a proximal gastrectomy for a KIT-positive gastric GIST. At 8 months after the gastric resection, the patient underwent a palliative resection for peritoneal metastases (more than 10 foci with the largest being 6.5 cm in diameter). At 11 months after initial resection, computed tomography (CT) revealed a solitary liver metastasis measuring 3.1 cm in diameter and multiple peritoneal metastases, of which the largest was 5.5 cm in diameter (Figure 1A

). The patient was referred to Niigata University Medical and Dental Hospital for imatinib treatment of the recurrent GISTs.

Imatinib treatment with four capsules of 100 mg imatinib mesylate (Glivec®, Novartis Pharma, Basel, Switzerland) once daily was started. After 3 months of imatinib treatment, abdominal CT scans showed that the liver and peritoneal metastatic tumours had reduced in size to 2.5 and 3.4 cm in diameter, respectively. Computed tomography scans conducted a further 3 months later showed that the antitumour effect of imatinib had continued, confirming a partial response (PR) (Figure 1B). However, 9 months after imatinib treatment was initiated, CT and magnetic resonance imaging (MRI) revealed disease progression at the metastatic site in the liver (Figure 1C). The peritoneal metastases had reduced to smaller lesions with an irregular shape and the intensity of soft tissue. Positron-emission tomography (PET) with [^18^F] fluorodeoxyglucose ([^18^F]FDG) as a tracer revealed increased uptake only in the liver metastasis (Figure 1D), suggesting that the liver tumour was active and insensitive to imatinib. At 12 months after the start of imatinib treatment, the relapsing tumour in the liver was excised with extended left hepatectomy. The patient is alive with no evidence of disease relapse 2 months after the liver resection.

## PATHOLOGICAL EXAMINATION

The resected liver specimen contained a metastatic GIST measuring 5.0 × 3.2 cm. The peripheral tumour showed a solid margin and there was central necrosis with cystic change. Viable spindle-shaped tumour cells were observed in the solid part by histology, and immunohistochemistry revealed that the tumour cells were KIT-positive. The proliferative activity of the tumour cells was approximately 80% in the regrowing focus, as evaluated by the Ki-67 labelling index (MIB1; Immunotech, Marseille, France).

## MUTATION ANALYSIS OF THE C*-KIT* GENE

RNA was isolated from fresh surgical specimens of liver metastatic tumours and cDNA synthesis was performed. The complete coding region of c-*kit* cDNA was directly sequenced using an ABI Prism 3100 Genetic Analyzer (Applied Biosystems, Foster City, CA, USA). The metastatic liver tumour had an in-frame mutation with a deletion of eight amino-acid residues corresponding to codons 552–559, and a substitutive isoleucine insertion in exon 11. An amino-acid substitution of codon 823-tyrosine (TAT) to aspartate (GAT) in exon 17 was also detected in the liver tumour. Mutation analysis of the c-*kit* sequence from the primary gastric GIST was then performed. Exons 11 and 17 of the c-*kit* gene were amplified by PCR using genomic DNA extracted from formalin-fixed, paraffin-embedded tissues as previously described (Taniguchi *et al*, 1999). Sequencing of the exon 11 and 17 c-*kit* PCR products revealed an in-frame deletion identical to that found in the metastatic liver tumour but no exon 17 mutation was found.

## DISCUSSION

Treatment failure with imatinib is a critical problem in patients with advanced chronic myeloid leukemia (CML) or metastatic GIST. Here, we present a case of metastatic GISTs showing late resistance to imatinib. DNA analysis revealed two gain-of-function mutations of the c-*kit* gene in the relapsing tumours: an in-frame mutation in exon 11, which encodes a region in the juxtamembrane domain; and a missense point mutation in exon 17, which encodes the tyrosine kinase (TK2) domain. A recent analysis of 112 metastatic GISTs by Heinrich *et al* (2003) revealed that c-*kit* mutations in exon 11 are the most common mutations found, with an incidence of 66.9%. In contrast, mutations in exon 17 were found in only two tumours (1.6%). In addition, this large-scale genetic study found no GIST with an activating mutation in more than one exon of the c-*kit* gene. Thus, our finding is significant in identifying two types of gain-of-function mutations simultaneously in one relapsing tumour focus. Moreover, the missense mutation in exon 17 was not found in the primary GIST of the stomach. This led us to conclude that the second mutation generated in the TK2 domain (Y823D) was responsible for the loss of sensitivity to imatinib in the metastatic tumours.

It is known that the mutational status of c-*kit* affects clinical response to imatinib in patients with metastatic GISTs (Heinrich *et al*, 2003). In patients with GISTs harboring exon 11 c-*kit* mutations, the PR rate was 83.5%, whereas patients with tumours containing an exon 9 mutation or no detectable mutation had PR rates of 47.8 and 0.0%, respectively. Exon 17 in the c-*kit* gene encodes the tyrosine kinase domain of KIT kinase, which suggests that substitution of an amino-acid residue in TK2 causes a critical conformational change that interferes with the effectiveness of imatinib. In CML, resistance to imatinib is conferred by a similar mechanism involving ABL kinase; this was shown clinically and *in vitro* (Gorre *et al*, 2001). However, data on the correlation between mutations in exon 17 and the patient's clinical response to treatment have been obtained from only two patients so far: one patient showed a partial response, while the other had a progressive disease. More data are needed to evaluate the clinical association of mutations in exon 17 of c-*kit* and sensitivity to imatinib, particularly in metastatic foci.
